# Prompt impact of first prospective statin mega-trials on postoperative lipid management of CABG patients: a 20-year follow-up in a single hospital

**DOI:** 10.1186/s12944-016-0292-6

**Published:** 2016-07-26

**Authors:** A. Palomäki, V. Hällberg, M. Ala-Korpela, P. T. Kovanen, K. Malminiemi

**Affiliations:** 1 Department of Emergency Medicine, Kanta-Häme Central Hospital, FIN-13530 Hämeenlinna, Finland; 2 Medical School, University of Tampere, Tampere, Finland; 3 University of Oulu, Institute of Health Sciences, Computational Medicine and Oulu University Hospital, Oulu, Finland; 4 School of Social and Community Medicine and Medical Research Council Integrative Epidemiology Unit, Computational Medicine, University of Bristol, Bristol, UK; 5 Wihuri Research Institute, Helsinki, Finland; 6 Department of Emergency Medicine, Tampere University Hospital, Tampere, Finland

**Keywords:** Coronary artery bypass, CABG, LDL cholesterol, Lipids, ApoB, Extended Friedewald, Statins, Statin intolerance

## Abstract

**Background:**

The long-term success of coronary artery bypass grafting (CABG) depends on secondary prevention. Vast evidence provided by the results of cholesterol mega-trials over two decades has shown that effective reduction of LDL cholesterol improves the prognosis of patients with coronary heart disease. However, the implementation of these results into the clinical practice has turned out to be challenging. We analysed how the information derived from clinical statin trials and international recommendations affected the local treatment practices of dyslipidaemia of CABG patients during a 20-year time period.

**Methods:**

The cohort includes all CABG patients (*n* = 953) treated in Kanta-Häme Central Hospital during the time period 1990–2009. At the postoperative visits in the cardiology outpatient clinic, each patient’s statin prescription was recorded, and blood lipids were determined.

**Results:**

During 1990–1994, 12.0 % of patients were on statins and during the following 5-year time periods the proportion was 57.2, 82.2 and 96.8 %, respectively. During the 20-year observation period (1990–2009), the effective statin dose increased progressively during these 5-year periods up to 36-fold, while the mean concentration of LDL cholesterol decreased from 3.7 to 2.1 mmol/l and that of apolipoprotein B from 1.3 to 0.8 g/l. In the very last year of follow-up, the mean concentrations of LDL-C and apoB were 1.83 mmol/l and 0.78 g/l, respectively. The most prominent increase in statin use and dosage took place during 1994–1996 and 2003–2005, respectively.

**Conclusions:**

Among CABG patients the lipid-lowering efficacy of statin therapy improved dramatically since 1994. This progress was accompanied by significant and favourable changes of lipid and apolipoprotein-B values. This study shows that it is possible to effectively improve lipid treatment policy once the results of relevant trials are available, and that this may happen even before international or national guidelines have been updated.

**Electronic supplementary material:**

The online version of this article (doi:10.1186/s12944-016-0292-6) contains supplementary material, which is available to authorized users.

## Background

Coronary artery bypass grafting (CABG) efficiently alleviates coronary heart disease (CHD) symptoms. However, without an effective secondary prevention, it does not offer permanent improvement of cardiac symptoms or prognosis [1, 2].

The primary target of the secondary prevention of coronary heart disease is effective reduction of low-density lipoprotein cholesterol (LDL-C), which diminishes cardiovascular and total mortality [3–6]. The lipid-lowering therapy is based on two approaches, i.e. change in lifestyle and pharmacological therapy [7]. In acute coronary syndrome patients it is desirable to start an effective statin therapy immediately after the event, i.e. already during the hospital stay [8].

According to early mega-trials, the turning points in secondary prevention of coronary heart disease were the years 1994, 1996 and 1997 when the first statin mega-trials 4S and CARE as well as the Post-CABG trial were published, and showed that effective therapy with statins improves the prognosis of patients with CHD [2–4]. The next key observations with a major influence on statin therapy occurred in 2004–2005, when the beneficial effects of intensive statin therapy, as compared with moderate statin therapy, were published [6, 8].

Two lipid-lowering recommendations, one in the U.S. and the other in Europe, were published just before the first statin mega-trials, i.e. in 1994 [9, 10]. They pointed out the central importance of increased cholesterol concentrations in determining the CHD risk; yet, due to the lack of evidence, the recommendations were conservative in their drug therapy guidelines [9, 10].

In 1998, the European targets for total cholesterol (T-C) and LDL-C were set below 5.0 mmol/l and 3.0 mmol/l, respectively [11]. One year later in the USA, the target for LDL-C in CABG patients was set below 100 mg/dL (2.6 mmol/l) [12]. Further in 2001 and 2003 LDL-C targets were set below 2.6 mmol/l and 2.5 mmol/l in the USA and Europe, respectively [13, 14]. Even though the goals were more demanding, the major emphasis was still on therapeutic lifestyle changes. Eventually, in 2004 the American guidelines and in 2007 the European guidelines for cardiovascular disease prevention recommended that in patients with established coronary heart disease LDL-C should be lowered below 2.5 mmol/l, or below 1.8/2.0 mmol/l, “if achievable” [15, 16].

The evidence indicates that the combined use of different apolipoprotein (apo) measures might improve the risk assessment of cardiovascular disease [17, 18]. Regarding the atherogenicity of the lipoproteins, the apolipoprotein B (apoB)-containing lipoproteins are of primary importance. Since one apoB molecule is present in each very-low-density lipoprotein (VLDL), intermediate-low-density lipoprotein (IDL) and LDL particle [19, 20], the plasma concentration of apoB indicates the total number of circulating atherogenic apoB-containing lipoprotein particles. ApoB concentration as a complementary measure was included first in recommendations in 2008 [7], and in the highest risk patients, the treatment goal was set to 0.80 g/l. To achieve this goal, effective lipid-lowering drug therapy is needed [21].

The aim of our study was to assess whether statin mega-trials and the recommendations based on their results influenced the local treatment practice regarding statin-dependent control of lipid levels in CABG patients over the critical 20-year period (1990–2009) which encompassed milestone publications regarding LDL-C lowering-based pharmacotherapy in preventive cardiology. We also attempted to learn whether the evolving changes in the LDL-C targets were reflected in the concentrations of different lipid classes and apolipoproteins in the patient cohort.

## Methods

### Patients

We studied all annual cohorts of CABG patients treated in Kanta-Häme Central Hospital (K-HCH) during the years 1990–2009. The primary catchment population of the hospital is 175 000 inhabitants living in Southern Finland. CABG was performed in Tampere University Hospital for 953 patients and, after the operation, they were transferred to K-HCH for further recovery.

After discharge the patients were followed according to hospital policy. Of the patients, 946 (99.3 %) had at least one visit in the outpatient clinic of cardiology during the first three postoperative months.

### Material analysis

Results of all patients who visited the outpatient clinic were analysed. Patients who had laboratory values on the first postoperative visit, but not on the 3-month visit, were also included; that is, a “last observation—carry forward” principle was adopted. Accordingly, data derived from the final postoperative outpatient visit are presented, 19 and 81 % of them being derived from the 1 to 3-month visits, respectively.

The year of CABG, postoperative lipid values, medication, and absence/presence of various CHD risk factors including smoking, hypertension, diabetes, and obesity (body mass index, BMI) were collected from the patient records. Patients’ data were coded, and data analysis was carried out with the coded material only. The study was approved by the Ethics Committee of Kanta-Häme Hospital District (Dnro E511/08).

The influence of the statin trials and recommendations on treatment goals were examined by comparing CABG patients’ postoperative lipid values taken at various time points over a time span of 20 years. The study period was divided into four consecutive 5-year time periods, namely 1990–1994, 1995–1999, 2000–2004, and 2005–2009. During this 20-year time period several new statins with different efficacies received their marketing authorisation. Accordingly, the daily dose and the dose-dependent LDL-C lowering effect of each individual statin had to be taken into account. This enabled us to compare the lipid-lowering efficacy of the statin therapy during the entire period.

A daily dose of 20 mg simvastatin or equipotent dose of another statin is the smallest statin dose used in most prospective trials, and this dose was defined as “daily statin dose index (DSDI) 1”. In the Tables and Graphs, in which the effective doses of various statins are presented, the DSDI 1 corresponds to a daily dose of 80 mg fluvastatin, 40 mg lovastatin or pravastatin, 20 mg simvastatin, 10 mg atorvastatin or 5 mg rosuvastatin. The lipid-lowering efficacy of each statin was assumed to be linearly correlated with the dose; i.e. the DSDI of 80 mg simvastatin, 40 mg atorvastatin or 20 mg rosuvastatin was 4 [22–25].

### Lipid analysis

Total and high-density lipoprotein cholesterol (HDL-C) concentrations were determined from plasma samples by using Hitachi 911 analyser with Boehringer-Mannheim reagents in 1990–1996. Roche Diagnostics enzymatic methods were used since 1996, which also applied to direct LDL-C analysis from 2000 onwards. All chemical analyses were carried out in the Laboratory of Kanta-Häme Central Hospital. Lipid analyses have been under the Nordic quality control during the entire study period.

For the calculation of LDL-C with the classical Friedewald formula (FW) data on T-C, HDL-C and triglycerides (TG) are required. Since FW is valid provided serum TG ≤ 4.5 mmol/l, we also applied a novel extended Friedewald (eFW) approach, which is more tolerant on elevated triglycerides [26, 27]. The eFW is based on artificial neural network regression algorithms which utilize data on classical FW inputs [26]. This method allowed us to calculate LDL-C, IDL cholesterol (IDL-C), HDL2 cholesterol (HDL2-C) and VLDL triglyceride (VLDL-TG) concentrations. It also computationally yields estimates of the apoB and apolipoprotein A-1 (apoA1) concentrations. To estimate HDL3 cholesterol (HDL3-C), the HDL2-C value obtained with eFW was subtracted from the measured HDL-C [26].

### Statistical analysis

Evolution of postoperative statin therapy and lipid values over 20 years was studied using ANOVA test in the 5-year groups. With dichotomal variables, the 5-year groups were compared using extended *χ*^2^ test [28]. D’Agostino’s test was used to determine normality and scedasticity. Percentage or mean and standard deviation of demographic characteristics and lipid variables are presented. Multiple linear regression analysis was used when comparing lipid values with each other, and also when LDL-C values obtained by enzymatic methods were compared with those obtained by the classical Friedewald equation or by the extended Friedewald approach.

## Results

Together 400 to 650 CABG patients were operated yearly in Tampere University Hospital during 1990–2009. On average, 50 patients of them were postoperatively treated annually in K-HCH. Patient records of altogether 946 subjects who attended postoperative cardiac outpatient clinic were analysed over a period of 20 years divided into four consecutive 5-year time periods, each consisting on average of 237 patients (ranging from 219 to 256 patients). The proportion of male patients ranged from 72 to 80 %. The changes in patients’ demographics followed both national and international trends, and the demographic characteristics are presented in Table 1.

**Table 1:** Demographics of patients undergone CABG during 1990–2009, divided into 4 consecutive 5-year periods

5-year period	1990–1994	1995–1999	2000–2004	2005–2009	Overall *p* ^a^
Number of patients	256	247	224	219	–
Proportion of males (%)	78	80	72	79	0.5193
Proportion of diabetics (%)	10	14	19	27	0.0122
Current and ex-smokers (%	64	55	54	56	0.4544
Age (years)	61.9 ± 7.7	64.0 ± 9.2	65.4 ± 9.4	66.5 ± 8.3	<0.0001
BMI (kg/m^2^)	26.5 ± 3.5	26.5 ± 3.5	27.2 ± 3.7	27.0 ± 4.1	0.0978
Systolic BP (mmHg)	149.6 ± 24.8	147.9 ± 23.2	137.8 ± 22.0	130.0 ± 20.4	0.0001
Diastolic BP (mmHg)	84.7 ± 11.6	82.1 ± 11.1	80.2 ± 9.8	77.5 ± 9.3	<0.0001

*Abbreviations*: *BMI* body mass index (kg/m^2^), *BP* blood pressure

^a^Extended *χ* ^2^ test and ANOVA were used for overall analysis. Percentage or mean ± standard deviation is presented

### Lipid treatment practice

Altogether 803 patients had their lipid values measured, 81.3 % of them at the 3-month visit. During the first 5-year period, 63.6 % of the patients and during the subsequent 5-year periods 85.6, 94.2 and 96.8 %, respectively, had their lipids analysed. Thus, there was an initial strong and statistically significant (*p* < 0.01) increase in the proportion of patients having their lipids determined.

**Fig. 1:**
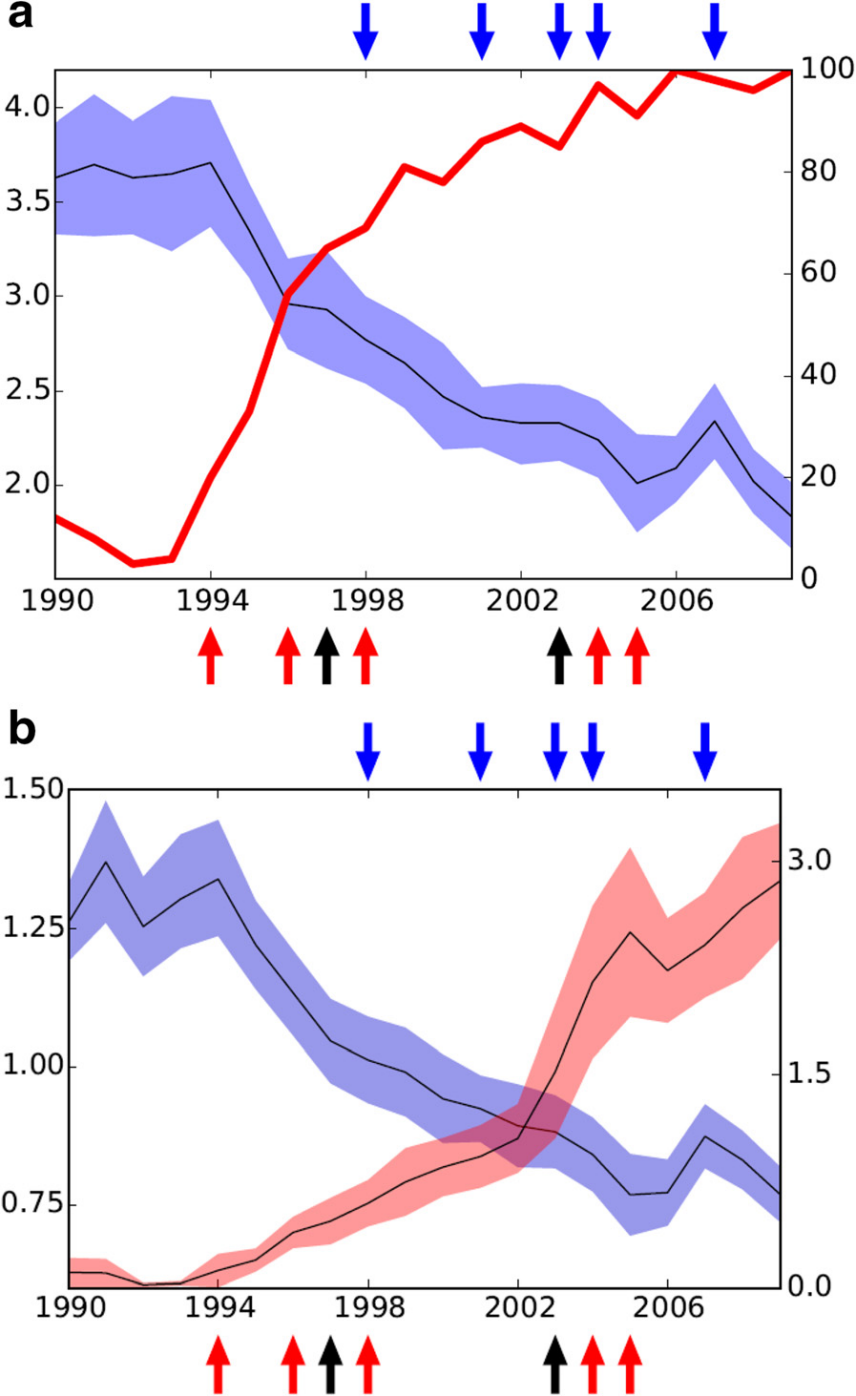
**a** LDL-C ± 1 SD calculated by Friedewald formula (mmol/l, black line ± 1 SD blue shadowing) and the amount of statin users (%, red line) at postoperative visits. **b** Plasma correlation of apoB ± 1 SD (g/l, black line ± 1 SD blue shadowing) and daily statin dose index (DSDI, black line ± 1 SD red shadowing) (see Methods) at postoperative visits. Red arrows indicate the publication of landmark studies from left to right: 4S^3^, CARE^4^, LIPID^5^, REVERSAL^6^ and PROVE IT^8^. Blue arrows refer to the publication of lipid lowering recommendations from left to right: Second European Task Force^11^, NCEP ATP III^12^, Third European Task force^13^, NCEP ATP III (Implication of recent clinical trials)^14^, and Fourth European Task Force^15^. Black arrows indicate the years, when the two most effective statins entered the Finnish market, i.e. atorvastatin in 1997 and rosuvastatin in 2003. LDL-C was calculated by Friedewald formula and apoB was obtained using extended Friedewald approach (see Methods)

During the 20-year observation period, the use of statins increased markedly, the most prominent increase having taken place during 1995–1999 (Additional file 1: Table S1B). Within the last years of the follow-up, practically all CABG patients were on statin therapy (Fig. 1a).

The DSDIs were small during the initial years, and effective statin doses increased progressively during the last years of follow-up. Thus, the average DSDIs of all patients, even when those without statin therapy were included, increased during four consecutive 5-year time periods from 0.07 (±0.23) to 0.46 (±0.53), 1.25 (±1.19) and 2.52 (±1.52) (p for trend < 0.001) (Fig. 1b, Table 2). The DSDI 2.86 (±0.41) during the very last year of observation corresponds to a mean simvastatin dose of 57 (±8) mg/day.

**Table 2:** Postoperative plasma lipid and lipoprotein levels in consecutive 5-year periods in statin-treated patients undergone CABG

5-year period	1990–1994	1995–1999	2000–2004	2005–2009	Overall *p* ^a^
DSDI	0.07 ± 0.23	0.46 ± 0.53	1.25 ± 1.19	2.52 ± 1.52	<0.0001
T-C (mmol/l)	5.70 ± 1.27	4.86 ± 1.00	4.20 ± 0.91	3.76 ± 0.82	<0.0001
LDL-C (mmol/l)^b^	3.70 ± 1.09	2.94 ± 0.85	2.35 ± 0.72	2.07 ± 0.64	<0.0001
HDL-C (mmol/l)	1.02 ± 0.29	1.12 ± 0.33	1.24 ± 0.32	1.22 ± 0.34	<0.0001
T-G (mmol/l)	2.20 ± 1.22	1.79 ± 0.96	1.56 ± 0.81	1.40 ± 0.65	<0.0001
Extended Friedewald approach (eFW)^c^					
VLDL-TG (mmol/l)	1.36 ± 0.85	1.12 ± 0.79	0.95 ± 0.6	0.85 ± 0.	<0.0001
IDL-C (mmol/l)	0.38 ± 0.14	0.28 ± 0.11	0.23 ± 0.11	0.23 ± 0.16	<0.0001
LDL-C (mmol/l)	3.55 ± 0.78	3.01 ± 0.71	2.49 ± 0.63	2.21 ± 0.51	<0.0001
HDL-2-C (mmol/l)	0.57 ± 0.23	0.67 ± 0.27	0.76 ± 0.27	0.77 ± 0.28	<0.0001
HDL-3-C (mmol/l)	0.46 ± 0.07	0.47 ± 0.07	0.48 ± 0.07	0.48 ± 0.07	0.0121
ApoA1 (g/l)	1.38 ± 0.22	1.38 ± 0.24	1.42 ± 0.24	1.37 ± 0.26	0.1864
ApoB (g/l)	1.30 ± 0.26	1.09 ± 0.26	0.90 ± 0.23	0.81 ± 0.18	<0.0001

Mean ± standard deviation are presented

*Abbreviations*: *DSDI* daily statin dose index, where the 1.0 corresponds the dose of simvastatin 20 mg per day (see Methods). In the intention to threat analysis all patients are included

^a^ANOVA was used for overall analysis

^b^LDL-C is determined using Friedewald calculation

^c^The eFW is based on artificial neural network regression algorithms which utilize data on classical FW inputs (see Methods)

### Plasma lipid and apolipoprotein levels

The mean concentrations of LDL-C were 3.7 and 2.1 mmol/l, and those of apoB 1.3 and 0.8 g/l, during the initial (1990–1994) and final (2005–2009) 5-year time periods, respectively (Fig. 1, Table 2). During the last single year of follow-up (2009), the mean concentrations of LDL-C and apoB were 1.83 mmol/l and 0.78 g/l, respectively.

**Fig. 2:**
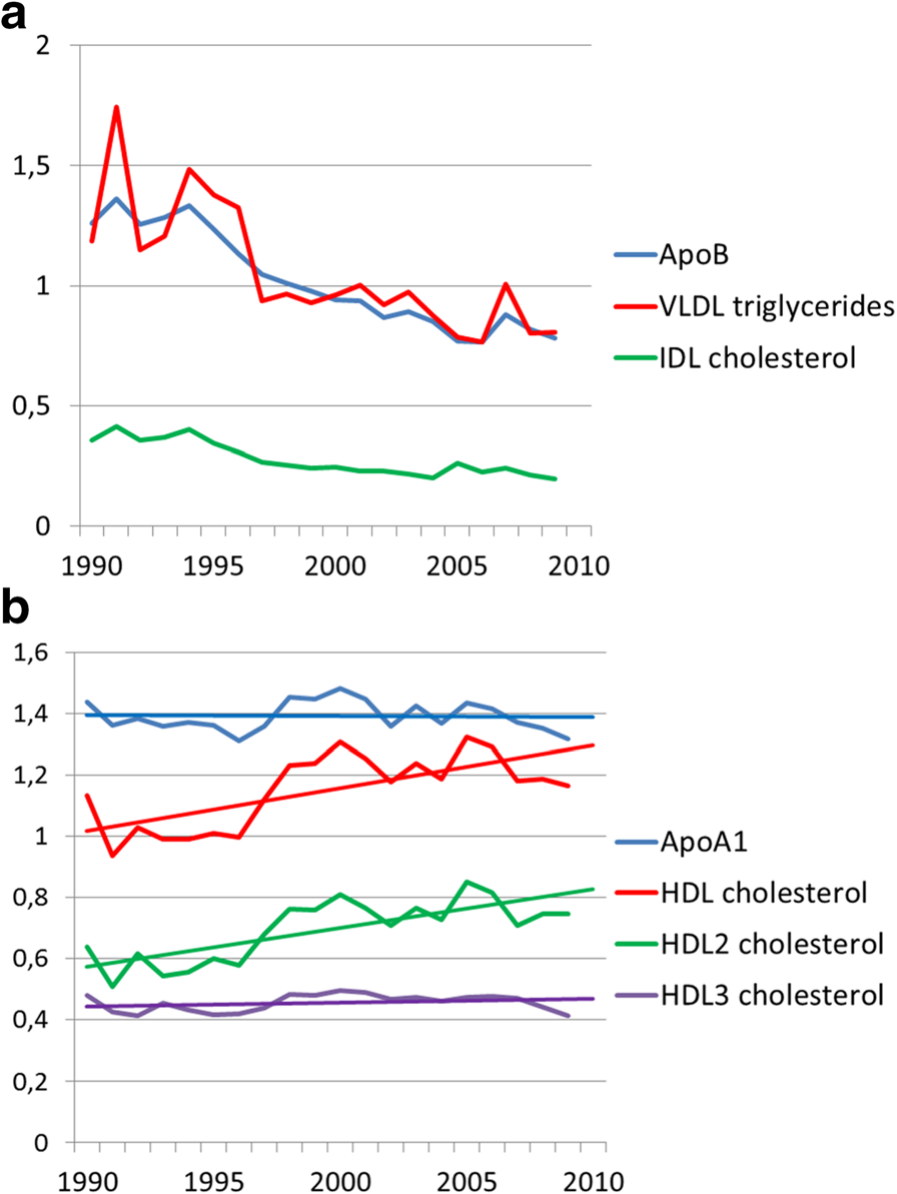
**a** Apolipoprotein B (ApoB, g/l), VLDL-triglycerides (mmol/l) and IDL-cholesterol (mmol/l) at postoperative visits. **b** Apolipoprotein A-1 (ApoA1, g/l), HDL-cholesterol (mmol/l), HDL2-cholesterol (mmol/l) and HDL3-cholesterol (mmol/l) at postoperative visits are shown both as curves and as linear representations. All parameters except HDL-C were obtained using extended Friedewald approach

The decrease in apoB concentration was progressive during the 20-year time span. It reflects not only the decrease in LDL-C concentration, but also in the concentrations of other apoB-containing lipoproteins, as seen in decreases of VLDL-TG and IDL-C, which were reduced proportionately (Fig. 2a). HDL-C increased from 1.02 (±0.29) to 1.22 (±0.34) particularly due to an increase in the concentration of HDL2-C, while the concentration of HDL3-C remained at a constant level of 0.45 mmol/l (Fig. 2b). The multiple linear correlations of different lipoproteins are presented in Additional file 2: Table S2B.

## Discussion

Our main findings indicate that during a 20-year follow-up (1990–2009) of CABG patients the following beneficial changes in lipid and lipoprotein measurements and in lipid treatment efficacies occurred in sequence. First, the percentage of patients with measured cholesterol levels increased, and thereafter, the share of patients on statin therapy and the efficacy of the therapy increased consecutively. Moreover, the concentrations of LDL-C and of apoB, the latter reflecting the number of atherogenic apoB-containing lipoprotein particles, decreased by 45 and 40 %, respectively.

Importantly, the primary changes took place immediately after the first statin mega-trial had been published, i.e. before their results had been integrated into the updated formulations of the relevant international guidelines [3, 4]. The mean dosage of statin increased rapidly after 2002, when the most effective statin, rosuvastatin, had become available in the market. In 2002 and 2005 DSDI-values were 1.08 and 2.56, respectively, see Fig. 1b). Although during the 20-year follow-up period, the levels of total cholesterol in the Finnish population steadily decreased [29], the decrease in our patients was 3- to 4-fold when compared with that of the general population. This finding accords with the notion that, at least the majority of the excess decrease in LDL-C among the CABG patients must have been due to statin treatment. Thus our results comply with the observed significant decreases in LDL-C found in mega-trials in which decreased cardiovascular morbidity and mortality with intensified statin therapy could also be achieved [3–6, 8].

The efficiency of secondary prevention of CHD in Europe has been evaluated in the Euroaspire studies I and II. In the Euroaspire II study (1999–2000) consisting of coronary patients (including also CABG patients) from 15 European countries, 42 % had their T-C < 5.0 mmol/l and 61 % were on lipid-lowering medication [30]. At the same time the corresponding numbers in our patient population were 68 and 76 %, respectively. In an international cross-sectional study by Gitt et al. [31], the prevalence of lipid abnormalities were assessed in 2008–2009 in more than 22 000 European and Canadian patients on statin treatment. In this study, 58 % of patients having a cardiovascular high-risk had their LDL-C < 2.5 mmol/l. Their mean DSDI was 1.85, corresponding to a simvastatin dose of 37 mg [31]. In another study carried out 2009–2010, out of 151 patients undergone CABG, 83.4 % had lipid profile measured during the first postoperative year [32]. In this group, the mean LDL-C level was 1.86 mmol/l, i.e. at the same level as it was during the last years of our study. It has recently been indicated, that physicians’ lipid-lowering treatment choices are usually more conservative than guideline recommendations [33]. However, our results reveal that, in a proactive clinic, it is feasible to react without delay to the inflowing relevant information describing results of new well-conducted lipid-lowering trials published in peer-reviewed high-impact journals.

The concentrations of HDL-C and phospholipids, as well as the HDL2/HDL3-ratio, increased in the CABG patients during the 20-year observational period. Regarding the potential clinical significance of these observations, following points need to be considered. The apparently beneficial changes must have been partly due to statin treatment and partly to life style changes. Inasmuch as beneficial life style changes are accompanied by beneficial changes in various cardiometabolic measures, quantitation of the role of changes in Apolipoprotein A-I containing lipoproteins or their components is not feasible. Similarly, the clinical relevance of the minor statin induced increases in various parameters of HDL particles and their constituents remains uncertain. Interestingly, the moderate increase in HDL-C levels with statins correlates with regression of coronary atherosclerosis [34]. With respect to the size of the HDL particles, the larger HDL2 particles have been associated with greater CHD protection than the smaller HDL3 particles [35, 36]. However in recent meta-analysis of 80 published investigations, no differences in cardioprotective properties between these two HDL subclasses were discerned [37, 38]. Importantly, regarding the cardioprotective functions of HDL, the scientific interest is shifting largely to unravelling the biologic activity of HDL [38]. Thus, assigning any presumed clinical significance to the modest increases in the various metrics of Apo A-I containing lipoprotein fractions is not possible at present.

Although LDL is considered the main atherogenic cholesterol-rich particle, also other apoB-containing lipoproteins contribute to intimal cholesterol deposition, i.e. they also are atherogenic [20]. Importantly, the recent increase in the incidence of metabolic syndrome and diabetes has re-emphasized the requirement for obtaining additional data on triglyceride rich lipoproteins like VLDL and IDL. For example, IDL particles and their cholesterol are contributing to the progression of coronary and carotid artery atherosclerosis [39]. Besides the number of apoB-containing lipoproteins, also the size of these particles is considered a key determinant in atherosclerosis [40]. The small dense LDL particles, when being present in high numbers, particularly increase the risk of ischemic heart disease [41]. While the large chylomicrons fail to penetrate the arterial wall, triglyceride rich chylomicron remnants and VLDL remnants (IDL) with smaller particle sizes, enter the arterial wall and so drive atherogenesis [20, 42, 43]. To the best of our knowledge, an observation of diminished IDL-C during intensified statin treatment with lipid values taken and analysed 1–3 months postoperatively over a time span of 20 years, has not been reported earlier.

A limitation associated with this study is its retrospective nature. Therefore we lack knowledge of possible statin intolerance, which is nowadays considered a notable problem in statin treatment. Although in observational studies even 10–15 % of patients have reported muscle symptoms, most of them might be successfully treated with careful management [44, 45]. During the last years of our study only about 2 % of patients were not on statins. That might represent the maximal percentage of patients suffering from clinically relevant statin intolerance in this well-motivated patient population. A possible explanation for the success in statin therapy might be the highly individualized care of the cardiovascular patients in our hospital.

Due to the retrospective nature of this study, the investigators had to rely on medical records with all their imperfections. For example, information concerning laboratory values and medications was occasionally incomplete. Nevertheless, for 85 % of the patients all relevant data were available. Moreover, during the last four decades our hospital has had a fairly stable catchment population without any significant national or international migration. Thus the genetic and racial backgrounds, two factors of potential importance considering the limited size of our study sample, have remained stable.

## Conclusions

To conclude, prominent increase of statin use in patients undergone CABG took place immediately after the first statin mega-trial was published. Further, over a 20-year period (1990–2009) the efficacy of statin treatment gradually increased and the concentration of LDL-C, measured 1–3 months postoperatively, gradually decreased. Also the decreases of apoB, IDL-C and VLDL-TG were favourable. Our results show that, based on the outcomes of relevant mega-trials, it is possible in a single centre to optimise clinical practice without delay even before they have led to reformulations of international guidelines.

## Additional files

Additional file 1: Table S1B. Percentages in consecutive 5-year periods of patients having statin therapy after the CABG during 1990–2009. (DOCX 14 kb) Additional file 2: Table S2B. Multiple linear regression analysis between lipid values obtained with different methods. (DOCX 16 kb)
