# Supplementary material for: Prompt impact of first prospective statin mega-trials on postoperative lipid management of CABG patients: a 20-year follow-up in a single hospital
Source: Lipids Health Dis. 2016 Jul 26;15:124. doi: 10.1186/s12944-016-0292-6 (PMC4962493; doi:10.1186/s12944-016-0292-6)
Supplement: Additional file 1: Table S1B. — Percentages in consecutive 5-year periods of patients having statin therapy after the CABG during 1990–2009. (DOCX 14 kb) [file 12944_2016_292_MOESM1_ESM.docx]

Additional file

**Table 1B.** Percentages in consecutive 5-year periods of patients having statin therapy after the CABG during 1990-2009

Statin / Years 1990–94 1995–99 2000–04 2005–09

Lovastatin (%) 9.3 13.6 8.9 0.5

Pravastatin (%) 0 6.4 10.2 0.9

Simvastatin (%) 2.7 29.6 31.1 52.3

Atorvastatin (%) 0 7.6 30.7 29.5

Rosuvastatin (%) 0 0 1.3 13.6

**Patients on statin (%) 12.0 57.2 82.2 96.8**
