# Supplementary material for: Prompt impact of first prospective statin mega-trials on postoperative lipid management of CABG patients: a 20-year follow-up in a single hospital
Source: Lipids Health Dis. 2016 Jul 26;15:124. doi: 10.1186/s12944-016-0292-6 (PMC4962493; doi:10.1186/s12944-016-0292-6)
Supplement: Additional file 2: Table S2B. — Multiple linear regression analysis between lipid values obtained with different methods. (DOCX 16 kb) [file 12944_2016_292_MOESM2_ESM.docx]

Additional file

**Table 2B**. Multiple linear regression analysis between lipid values obtained with different methods

|  | **Enzymatic methods** | | | | | | | | | | | |  |  |
| --- | --- | --- | --- | --- | --- | --- | --- | --- | --- | --- | --- | --- | --- | --- |
|  | T–C | | | LDL-C | | | HDL-C | | | TG | | |  |  |
| **Enzymatic methods** | | |  | | |  | | |  | | |  | | |
| Total cholesterol | | 1.000 | | | 0.936 | | | 0.083 | | | 0.459 | | |  |
| HDL cholesterol | | 0.083 | | | -0.086 | | | 1.000 | | | -0.359 | | |  |
| Triglycerides | | 0.459 | | | 0.292 | | | -0.359 | | | 1.000 | | |  |

**Friedewald calculation**

| LDL-C | 0.933 | | 0.985 | | -0.091 | | 0.254 | |
| --- | --- | --- | --- | --- | --- | --- | --- | --- |
| **Extended Friedewald**  **approach (eFW)*** |  |  | |  | |  | |  |
| LDL cholesterol | 0.921 | 0.966 | | 0.027 | | 0.155 | |  |
| HDL2 cholesterol | 0.013 | -0.134 | | 0.987 | | -0.449 | |  |
| HDL3 cholesterol | 0.332 | 0.125 | | 0.772 | | 0.095 | |  |
| VLDL triglyceride | 0.404 | 0.238 | | -0.383 | | 0.995 | |  |
| IDL cholesterol | 0.720 | 0.656 | | -0.177 | | 0.618 | |  |
| ApoA1 | 0.461 | 0.240 | | 0.887 | | 0.042 | |  |
| ApoB | 0.890 | 0.848 | | -0.320 | | 0.728 | |  |

Multiple linear correlation coefficients are shown. * The extended Friedewald approach (eFW) is based on artificial neural network regression algorithms which utilize data on classical Friedewald (FW) inputs (see Methods).
